# Rectum Dose Constraints for Carbon Ion Therapy: Relative Biological Effectiveness Model Dependence in Relation to Clinical Outcomes

**DOI:** 10.3390/cancers12010046

**Published:** 2019-12-21

**Authors:** Kyungdon Choi, Silvia Molinelli, Stefania Russo, Alfredo Mirandola, Maria Rosaria Fiore, Barbara Vischioni, Piero Fossati, Rachele Petrucci, Irene Turturici, Jon Espen Dale, Francesca Valvo, Mario Ciocca, Andrea Mairani

**Affiliations:** 1 Clinical Department, Centro Nazionale di Adroterapia Oncologica, 27100 Pavia, Italy; kyungdon.choi@cnao.it (K.C.); silvia.molinelli@cnao.it (S.M.); Stefania.Russo@Cnao.it (S.R.); Alfredo.Mirandola@Cnao.it (A.M.); MariaRosaria.Fiore@Cnao.it (M.R.F.); Barbara.Vischioni@Cnao.it (B.V.); Rachele.Petrucci@Cnao.it (R.P.); Francesca.Valvo@Cnao.it (F.V.); mario.ciocca@cnao.it (M.C.); 2 Department of Physics, University of Pavia, 27100 Pavia, Italy; 3 Medical Department, MedAustron Ion Therapy Center, 2700 Wiener Neustadt, Austria; Piero.Fossati@medaustron.at; 4 Department of Diagnostic Imaging, Molecular Imaging, Interventional Radiology and Radiotherapy, University of Roma Policlinico TorVergata, 00133 Roma, Italy; ireneturturici@alice.it; 5 Department of Oncology and Medical Physics, Haukeland University Hospital, 1400 Bergen, Norway; jon.espen.dale@helse-bergen.no; 6 BioPT Group, Heidelberg Ion-Beam Therapy Center, 69120 Heidelberg, Germany

**Keywords:** carbon ion therapy, RBE modeling, rectum constraints, FRoG

## Abstract

The clinical application of different relative biological effectiveness (RBE) models for carbon ion RBE-weighted dose calculation hinders a global consensus in defining normal tissue constraints. This work aims to update the local effect model (LEM)-based constraints for the rectum using microdosimetric kinetic model (mMKM)-defined values, relying on RBE translation and the analysis of long-term clinical outcomes. LEM-optimized plans of treated patients, having suffered from prostate adenocarcinoma (*n* = 22) and sacral chordoma (*n* = 41), were recalculated with the mMKM using an in-house developed tool. The relation between rectum dose-volume points in the two RBE systems (D_LEM|v_ and D_MKM|v_) was fitted to translate new LEM-based constraints. Normal tissue complication probability (NTCP) values, predicting late rectal toxicity, were obtained by applying published parameters. No late rectal toxicity events were reported within the patient cohort. The rectal toxicity outcome was confirmed using dosimetric analysis: D_MKM_VHs lay largely below original constraints; the translated D_LEM|v_ values were 4.5%, 8.3%, 18.5%, and 35.4% higher than the nominal D_MKM|v_ of the rectum volume, v—1%, 5%, 10% and 20%. The average NTCP value ranged from 5% for the prostate adenocarcinoma, to 0% for the sacral chordoma group. The redefined constraints, to be confirmed prospectively with clinical data, are D_LEM|5cc_ ≤ 61 Gy(RBE) and D_LEM|1cc_ ≤ 66 Gy(RBE).

## 1. Introduction

The physical and radiobiological characteristics of carbon ion beams, i.e., finite range, inverse depth dose profile, sharp lateral penumbra and increased relative biological effectiveness (RBE) at the end of range, make them suitable and potentially advantageous for the treatment of tumors that are radio-resistant to conventional radiotherapy and/or in close proximity to critical organs at risk (OARs) [1]. Comparing clinical results obtained with carbon ion radiotherapy (CIRT) at different institutions is, however, not always straightforward—as the reported nominal RBE-weighted doses depend critically on the different RBE models used [2,3,4].

The Italian National Hadrontherapy Center (CNAO, Pavia, Italy) employs fractionation schedules—derived from clinical experience acquired at the National Institute of Radiological Science (NIRS, Chiba, Japan)—for prostate adenocarcinoma (AdC) and sacral chordoma treatments, while changing the prescription dose to account for the use of different models for plan optimization [3,5]. Particularly, the local effect model (LEM—version I) is employed by European centers [6,7], while Japanese centers use either the semi-phenomenological mixed-beam model [8], or the modified microdosimetric kinetic model (mMKM) [9]. These latter two models have been clinically validated at the NIRS for their consistency and both will be referred to here as mMKM [10].

For the treatment of tumors in the pelvis, radiation-induced rectal damage is a major issue impacting quality of life during and after treatment [11,12]. In conventional radiation therapy, several studies concerning rectum tolerance doses and normal tissue complication probability (NTCP) parameters are available [11,13]; by contrast, CIRT is a fairly novel modality and long-term rectal toxicity data are still under investigation. Despite well-known differences in the two RBE systems outside the target volume, CNAO is following a conservative approach, by prudentially adopting rectum constraints from the experience acquired through NIRS [14,15]. The late rectal toxicity of prostate AdC patients treated at NIRS was recently reported and fitted to the Lyman–Kutcher–Burman (LKB) NTCP model [16]. Additionally, a new study showed that the dose to 2 cc of the rectum volume (D_2cc_) could represent a good predictor of CIRT rectal damage [17].

A fast-forward dose calculation system (FRoG), running on graphics processing units, capable of calculating both LEM and mMKM RBE-weighted doses (D_LEM_ and D_MKM_) for the same treatment plan, was developed in a collaboration between CNAO and the Heidelberg Ion Therapy Center (HIT, Heidelberg, Germany) [18,19]. In this work, FRoG was used to compute and analyze differences between LEM and mMKM RBE-weighted dose distribution (D_RBE_) to the rectum for prostate AdC and sacral chordoma patients previously treated at CNAO. In parallel, we analyzed clinical outcomes from CNAO in relation to the NIRS rectum toxicity, and applied the NIRS NTCP model [16] to evaluate the expected probability of late rectal damage based on a patient cohort with a longer follow-up period. The main aim was to establish and propose updated LEM-based planning constraints for safe clinical application, correcting the over-conservative approach adopted since the beginning of clinical activity at CNAO. 

## 2. Results

**Figure 1:**
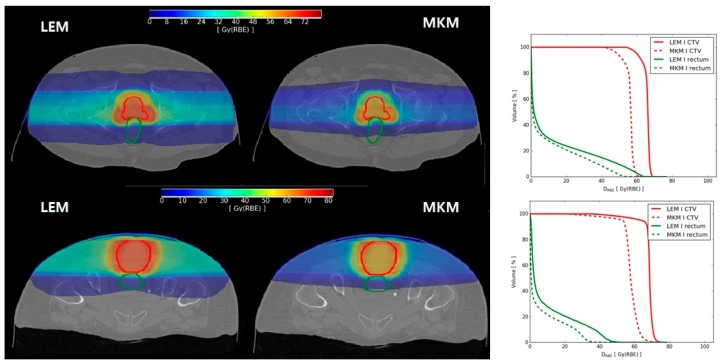
Left side: axial view of (upper panel) prostate and (lower panel) sacral chordoma patients local effect model (LEM) and microdosimetric kinetic model (mMKM) RBE-weighted dose distributions with clinical target volume (CTV—red line) and rectum (green line) contours. Right side: corresponding dose volume histograms (DVHs) for LEM RBE-weighted dose (D_LEM)_ (solid line) and mMKM RBE-weighted doses (D_MKM_) (dotted line).

Two representative cases, one of prostate AdC and one of sacral chordoma, are shown in Figure 1. Together with the axial view of D_RBE_ distributions, we also plotted the corresponding clinical target volume (CTV) and rectum dose volume histograms (DVHs), to highlight differences between the two RBE systems. In our clinical practice, dose deviations between LEM and mMKM in the CTV have been partially accounted for [3,5]; while no correction was applied until recently for dose over-estimation in OARs [15].

The best model fitting the relation between LEM and mMKM D_v_ was a quadratic regression:

D_MKM|v_ = a × (D_LEM|v_)^2^+ b × D_LEM|v_ + c, (1)

**Figure 2:**
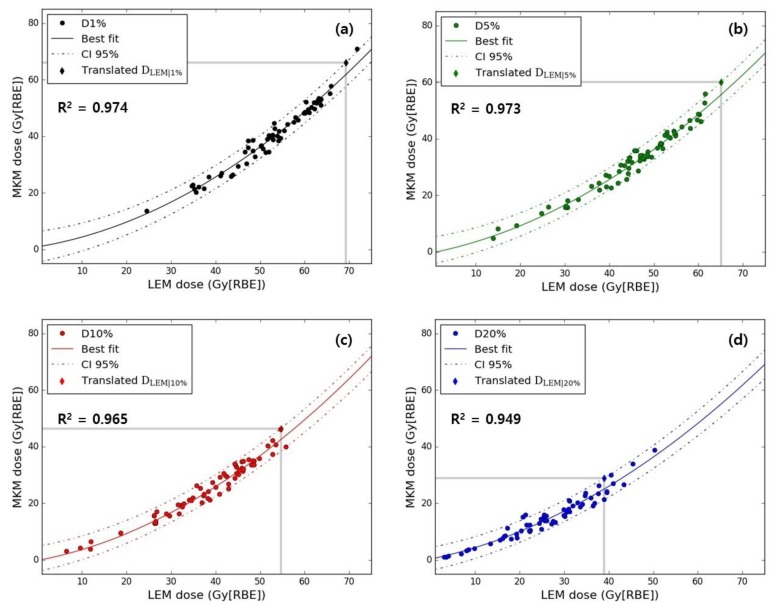
Rectum D_MKM_ as a function of D_LEM_ for (**a**) D_1%_, (**b**) D_5%_ (**c**) D_10%_ and (**d**) D_20%_ are presented with the corresponding fitting functions (solid lines represent the best fits and dashed lines the 95% confidence interval (CI)). Coefficients of determination (R^2^) for each parameter are given as well. In each plot, grey lines indicate the original D_MKM|v_ constraint and corresponding D_LEM|v_ translation (diamond).

Based on the applied model, the translation of the original NIRS D_MKM|v_ to new D_LEM|v_ values was made possible: 42.9 Gy(RBE) (confidence interval (CI) 95%: 38.9–46.8), 57.7 Gy(RBE) (54.7–61.0), 68.2 Gy(RBE) (65.1–70.8) and 72.0 Gy(RBE) (69.3–74.8) for v—20%, 10%, 5% and 1%, respectively. In Figure 2, patient-specific D_MKM|v_ values are plotted as a function of D_LEM|v_ for v—20%, 10%, 5% and 1% of the rectum volume, together with the best fit and corresponding 95% CI.

Lower bound CI values will be considered as newly translated D_LEM|v_ values. Using the lower bound CI, we aimed to take into account the sources of uncertainty involved in the described analysis.

**Figure 3:**
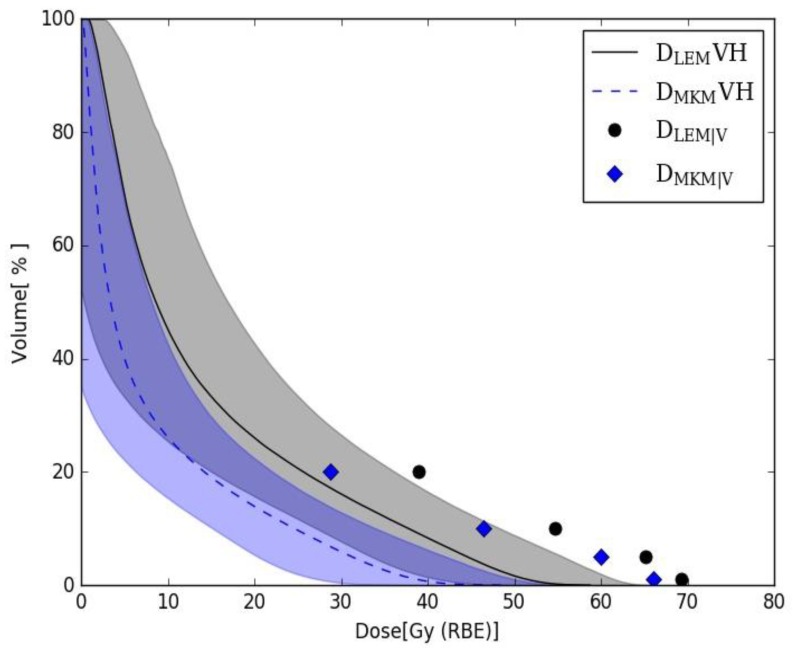
Average D_LEM_ (black solid line—grey band) and D_MKM_ (blue dotted line— blue band) rectum DVHs with D_MKM|v_ (blue diamonds) and translated D_LEM|v_ (black dots) values. DVH bands represent ± 1 standard deviation.

Averaged D_LEM_ and D_MKM_ rectum DVHs over the selected patient cohort are shown in Figure 3. The DVH bands represent ± 1 standard deviation.

The prostate AdC and sacral chordoma delivered treatment plans were optimized using LEM-based, RBE-weighted doses while applying D_MKM|v_ constraints. As shown in Figure 3, the average D_LEM_ rectum DVH lies under D_MKM|v_ constraints; with a portion of patient specific DVHs exceeding nominal values.

More specifically, D_LEM_VHs exceed the D_MKM|20%_ constraint of 28.8 Gy(RBE) in 42.9% of the cases (20 of the sacral chordoma and 7 prostate AdC patients); the D_MKM|10%_ of 46.4 Gy(RBE) was exceeded in 1.6% of the patients (one prostate AdC case); the D_MKM|5%_ constraint of 60 Gy(RBE) was exceeded in 3.2% of the cases (two prostate AdC case); no case exceeded the D_MKM|1%_, of 66 Gy(RBE).

In clinical practice, the constraints for OARs are compromised when priority is given to target coverage. No increase in complication rate was determined by the high percentage of D_LEM_VHs exceeding D_MKM|20%_. On the other side, only 3.2% (two prostate AdC cases) of D_MKM_VHs exceeded D_MKM|20%_. When relating to the translated D_LEM|v_ constraints, the percentage of non-compliant D_LEM_VHs drastically decreased to 3.2% for D_LEM|20%_ (consisting in the same two prostate AdC patients resulting from the mMKM analysis), and 0% for the other volume points, in perfect agreement with the mMKM scenario.

A total of five patient plans (2 prostate cases, 2 L_SAC_ cases and 1 H_SAC_ case) were re-optimized with translated D_LEM|v_ values and re-calculated with FRoG to analyze the rectum D_MKM_VHs. Table 1 presents both D_LEM|v_ and D_MKM|v_ (v: 20%, 10%, 5%, 1% of the rectum volume) values for the newly optimized plans, to be compared with the reference constraints, expressed in the corresponding RBE language. From the recalculation of mMKM RBE-weighted dose distributions for the five plans, we demonstrated that nominal D_MKM|v_ constraints were still respected, confirming the predictive power of the adopted regression model. D_20%_ and D_10%_ were not applied during optimization of the sacral chordoma plans and, therefore, values exceeded the constraints in both LEM- and mMKM-based scenarios.

**Figure 4:**
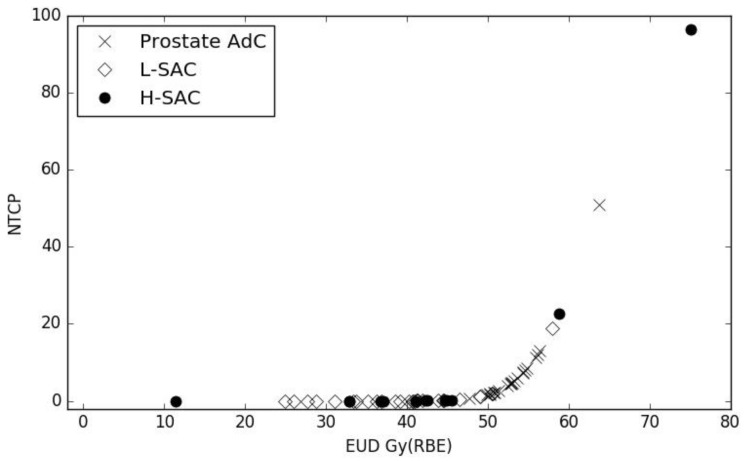
Late rectal toxicity normal tissue complication probability (NTCP) values as a function of rectum equivalent uniform doses (EUD) for prostate adenocarcinoma (AdC) (cross), L_SAC_ (empty diamonds) and H_SAC_ (full circles) patients.

Equivalent uniform doses (EUDs) and the corresponding NTCPs were calculated from each patient’s D_MKM_ distribution with FRoG, using G1 toxicity parameters reported by Fukahori et al., and are presented in Figure 4.

The average EUDs were 53.3 Gy(RBE) (CI 95% 52.0–54.6), 38.2 Gy(RBE) (35.2–41.2) and 39.8 Gy(RBE) (31.9–47.7) for prostate, L_SAC_ and H_SAC_ groups, while the corresponding NTCP values were 5.23% (CI 95% 3.41–7.85), 0.00% (0.00–0.02), and 0.01% (0.00–0.62), respectively. Lower EUD and NTCP values reported for sacrum cases result from the stricter constraints applied during plan optimization. The resulting average value for the NTCP is in agreement with early clinical outcomes: over all the prostate AdC and sacral chordoma patients treated at CNAO, no rectal complication higher than G0 was reported at follow-up longer than 6 months. One patient from the H_SAC_ group showed a very high NTCP for late rectal toxicity (96.4%). This patient had a large planning target volume (3800 cc) compressing the rectum (144 cc) for the whole extent of the posterior rectal wall, widely involved in the high dose treatment area. However, it should be noted that the patient’s last follow-up was at 20 months, shorter than the minimum follow-up of patients included in the Japanese NTCP study (>36 months).

## 3. Discussion

Dose constraints for OARs in CIRT are not yet completely established. To take advantage of the long-term experience of NIRS, where patients have been treated with carbon ions since 1994 [20], we adopted the same fractionation schemes as NIRS for most clinical protocols, while modifying prescription doses to account for D_RBE_ dependence based on the RBE model [3,5]. In contrast, the optimization constraints for several OARs (e.g., optic structures, brainstem and rectum) were conservatively taken from Japanese clinical practice [21] without any correction. This approach could eventually compromise target coverage and result in a sub-optimal treatment. After 5 years of clinical experience based on evaluation of plan robustness, optimization of adaptive protocols and analysis of normal tissue toxicities, we could redefine LEM-based constraints for the optic pathways [15] and brainstem. In this work, we used the same approach to update and harmonize rectum constraints for all treatments based on a 16-fraction schedule.

Observed rectal toxicity among pelvic patients treated at CNAO was very low. According to the Common Terminology Criteria for Adverse Events version 4.03 (CTCAE) (U.S. Department of Health and Human Services, Washington, DC, USA) [22] scale, from the prostate AdC group, one case of rectal bleeding G1 was reported at three months of follow-up and resolved by the ninth month of follow-up. Concerning sacral chordoma patients, three cases of acute proctitis G1 were reported during treatment. Of these, one patient was lost at follow-up and the other two cases of gastrointestinal (GI) disorder were resolved at first follow-up. Concerning late complications, no bleeding nor signs of rectal wall damage were observed. As reported in the results section, D_MKM_VHs lie largely below original MKM constraints, as demonstrated by the promising results for clinical outcome reported to date regarding rectal damage.

When comparing dose quantities calculated with a different treatment planning system (TPS), uncertainties must be taken into account affecting both absorbed and RBE-weighted dose distributions [5]. The expected differences between NIRS and CNAO dose computation systems are, in part, the rationale underlying the over-conservative, no-correction approach applied to OARs constraints at the beginning of clinical activity at CNAO. Both Syngo and FRoG were validated against the Fluka-MC code [18,23] to guarantee dosimetric accuracy and, along with that, dosimetric agreement between the two systems. Finally, the translation of the D_RBE|v_ values was performed in the same calculation frame (using FRoG) and was therefore not affected by the previously mentioned sources of dose deviation. Ultimately, the goal of deriving new constraints was to improve treatment quality in terms of tumor control, with a negligible increase in healthy tissue toxicity, and therefore, the implementation of these constraints to clinical practice must be performed with caution.

Also, uncertainties must be accounted for when applying NTCP parameters to a patient population different from the one they were optimized for [24]. For sacral chordoma patients, we applied an NTCP model based on prostate AdC patient toxicity outcomes, on the grounds that the rectum dose was delivered in the same number of fractions. Dose deviations between the two calculation systems from which the DVHs and EUD values were extracted could affect the NTCP results. In addition, the Japanese patient follow-ups were significantly longer (>36 months) than the follow-ups for our patient cohort. Taking these uncertainties into account, the estimated probability of late rectal complications was very low. In Okonogi et al., D_MKM|2cc_ was found to be the most significant predictive factor for late rectal morbidities, with a threshold of 57.3 Gy(RBE) to limit the risk for a 20 fraction treatment schedule [17]. Under the hypothesis that schedules could be compared based on the biological effective dose (BED) concept, the corresponding 16 fraction threshold value, assuming an α/β ratio of 3.9 Gy for the rectum [25], would be 53.5 Gy(RBE). Over our patient cohort, the average D_MKM|2cc_ was (34.7 ± 11.1) Gy(RBE) with only one H_SAC_ patient exceeding the 16 fraction threshold (D_MKM|2cc_ = 55.8 Gy(RBE)). This last case corresponds to the highest NTCP value described in the results section.

From NIRS NTCP analysis, the rectum appears as a serial organ regarding late complications, in agreement with conclusions from Okonogi et al. [17] Therefore, in the definition of updated constraints, focus was given to high rectum doses. The new values were expressed in cc to overcome the previously discussed differences determined by rectum size variation.

**Figure 5:**
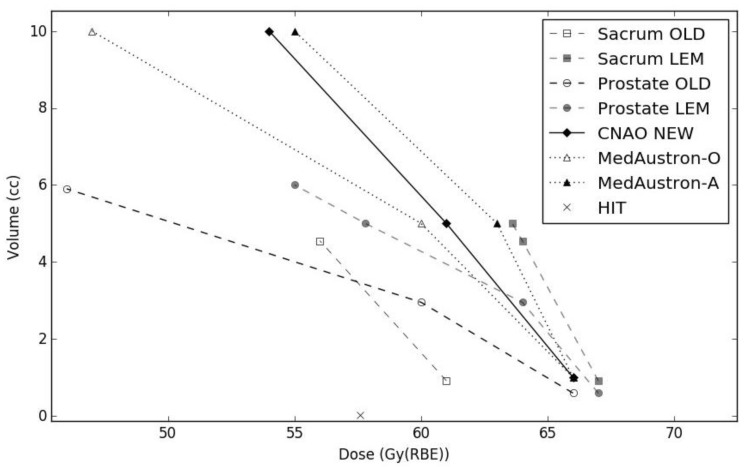
Rectum dose-volume constraints: old clinically applied values (Sacral chordoma—open square; prostate AdC—open circle), LEM-translated (Sacral chordoma—full square; prostate AdC full circle), new clinically defined (full diamond), MedAustron optimal (open triangle), MedAustron acceptable (full triangle), Heidelberg Ion-Beam Therapy Center (HIT) (cross).

Based on the results presented in this work, for all future pelvic patient plan optimizations receiving a 16 fraction schedule, we set new rectum constraints to D_1cc_ ≤ 66 Gy(RBE) and D_5cc_ ≤ 61 Gy(RBE). A limit of D_10cc_ ≤ 54 Gy(RBE) will also be applied, without compromising target coverage. In Figure 5, new constraints are plotted together with the previously applied D_MKM|v_ and the translated D_LEM|v_, where volume percentages have been translated to absolute values from the average rectal volume of the prostate AdC and sacral chordoma groups, respectively. The new D_1cc_ constraint will, on average, lie below the lower CI bound of translated D_LEM|v_ for both patient groups; while D_5cc_ has been established as the mean value of average extrapolated D_LEM|v_ for prostate and sacrum cases (also plotted in Figure 5), at the corresponding volume size of 5 cc. These constraints lie inside the range of protocol values defined at MedAustron (Wiener–Neustadt, Austria) for the CIRT of pelvic tumors, based on the same fractionation scheme in use at CNAO. Two particular sets of constraints are in clinical use: a lower dose level defined as optimal (MedAustron–O), and a higher dose level defined as acceptable (MedAustron–A). Uhl et al. reported HIT plan optimization constraints for patients with sacro-coccygeal chordoma, treated with a 16-fraction CIRT schedule [25]. The authors defined a structure corresponding to the overlap of the planning target volume and the rectum, to which a maximum dose constraint of 57.6 Gy(RBE) was assigned.

## 4. Materials and Methods

### 4.1. Patient Treatment and Follow-Up

A total of 63 representative patients, treated for a tumor in the pelvis, were selected for this study: 22 prostate AdC cases, and 41 sacral chordoma cases. High-risk prostate AdC patients were consecutively treated between July 2013 and June 2017, with a dose prescription of 66.4 Gy(RBE) and results analyzed after a median follow-up time of 33 months (range: 14–62). In the analysis, 28 additional sacral chordoma cases treated with 70.4 Gy(RBE) (L_SAC_), and 13 additional cases treated with 73.6 Gy(RBE) (H_SAC_) between April 2013 and July 2018, were included, with a median follow up time of 39 months (range: 12–72) and 20 months (range: 8–70) for the L_SAC_ and H_SAC_ populations, respectively.

For treatment simulation and delivery, patients were positioned on a personalized cushion and immobilized with a solid thermoplastic mask, to optimize set-up reproducibility and minimize inter- and intra-fraction uncertainties. The patient set-up verification makes use of two orthogonal X-ray acquisitions for the registration of the bone anatomy, as well as a cone-beam CT (computed tomography)-scan for organ filling control. All patients received the CIRT in 16 fractions, 4 days-a-week, delivered with a pencil beam scanning technique. Prostate AdC patients were treated in a supine position, with two lateral-opposed fields, while sacral chordoma patients were treated in prone position with a minimum of three fields (two lateral-opposed and one vertical), unless for completely lateralized lesions. Periodic re-evaluation CT-scans were planned for every two weeks of treatment (8 fractions), except for in the case of patient-specific clinical indications.

The rectum volume was defined using the planning CT-scan, according to the Radiation Therapy Oncology Group (RTOG) contouring guidelines, starting inferiorly from the lowest level of the ischial tuberosity (right or left). Contouring ended superiorly before the rectum lost its round shape in the axial plane and connects anteriorly with the sigmoid [26].

All the rectum contours were independently reviewed by two medical doctors for compliance with guidelines.

The mean rectum volume was (80 ± 36) cc over the whole patient population, with an average of (59 ± 15) cc for prostate and (91 ± 39) cc for sacrum cases. The average rectum volume size is affected by the different preparations patients undergo—according to the specific treatment protocol—and by clinical baseline conditions. The prostate AdC patients were routinely prepared with an enema before imaging and treatment, whereas no specific measure was employed for sacral chordoma patients. Moreover, rectum volumes were generally larger in the latter group due to tumor involvement with the sacral nerves causing the relaxation of the rectal wall.

Currently applied rectum dose constraints for a 16 fraction prostate AdC treatment are: dose to volume percentage v—5% and 1% (D_v_) lower than 60 and 66 Gy(RBE), respectively. In addition, D_v_ limits are set to minimize rectum lower doses to 20% and 10% of the volume, to 28.8 Gy(RBE) and 46.4 Gy(RBE), respectively, which can be compromised to prioritize target coverage. These D_v_ values fit a rectum upper boundary D_MKM_VH, provided by NIRS colleagues, routinely taken as a reference for prostate AdC plan approval. In consideration of the larger rectum volumes involved, and considering the lower reproducibility in the rectum filling, shape and position of sacral chordoma patients, the D5% and D1% described values were applied to a planning risk volume, obtained with a uniform 5 mm expansion of the rectum. Rectum constraints were therefore slightly lower: D5% ≤ 56 Gy(RBE) and D1% ≤ 61 Gy(RBE). This approach was conceived with the aim of accounting for anatomical changes which could only be detected with the aid of periodic re-evaluation CT scans. D20% and D10% were not considered for the plan optimization of sacral chordoma cases.

Follow-up visits for the prostate AdC patients are scheduled at three, six and twelve months after treatment completion; every six months for the following two years and yearly afterwards, until the fifth year from the treatment. The patients will undergo a clinical examination, with a three-monthly evaluation of prostate-specific antigen and testosterone levels. A magnetic resonance imaging (MRI) scan and uroflowmetry will also be performed yearly.

Sacral chordoma patients will undergo follow-up visits, every three months for the first two years, and every six months for the following three years, with a clinical examination and an MRI scan. A thorax CT scan and whole spine MRI will be performed annually.

Gastrointestinal (GI) complications were scored according to the GI disorders terms of the CTCAE. In Fukahori et al. [16], GI complications were scored according to the guidelines of the Radiation therapy Oncology Group (RTOG) and the European Organization for Research and Treatment of Cancer (EORTC) [27]. With the aim of applying NIRS NTCP parameters to CNAO rectum DVHs, the toxicity originally reported according to CTCAE was re-scored for this analysis using the RTOG scale.

Patients analyzed in the paper cannot be identified as all the research has been conducted with anonymous data. All patients enrolled in the clinical trials gave their free informed consent to the treatment and the use of their data for research purposes. All the patients enrolled at CNAO freely signed their consent to the treatment and to the management of their clinical data, in anonymized way, for research purposes.

Most of the clinical cases enquired were enrolled in clinical trials:

▪ CNAO S16/2012C Approved by referral Ethics Committee “CNAO” on 19 December 2012, “Phase II clinical trial on high risk prostate cancer treated by carbon ions radiation therapy”.
▪ CNAO S13/2012/C Approved on 21 December 2016, by referral Ethics Committee “Area Pavia”, “Phase II clinical trial on trunk sarcoma (of bones and of soft tissues) treated by carbon ions radiation therapy”.
▪ CNAO 33 2016 C “Sacral Chordoma: a Randomized & Observational study on surgery versus definitive radiation therapy in primary localized disease (SACRO)” approved on 24 November 2016, by referral Ethics Committee “Area Pavia”.

### 4.2. Dose Recalculation and Analysis

All the treatment plans for the patient group treated in the pelvic region were calculated for clinical purposes with the Syngo PT (Siemens AG healthcare, Erlangen, Germany) TPS. CT image, RTdose, RTstructure and RTplan DICOM files were exported from Syngo and imported in FRoG for the recalculation of D_LEM_ and D_MKM_.

From the two corresponding rectum DVHs, patient-specific D_MKM|v_ values (v: 1%, 5%, 10%, 20%), were extracted and plotted as a function of the corresponding D_LEM|v_. New D_LEM|v_ values translated from the original D_MKM|v_ constraints were determined from these plots. The regression was performed with Scipy libraries providing numerical integration, interpolation, optimization, linear algebra and statistics [28], and the software IBM SPSS Statistics for Windows, Version 26.0 (IBM Corp., Armonk, NY, USA). The average rectum DVHs over the patient population, in the two RBE systems, were evaluated in relation to original D_MKM|v_ and translated D_LEM|v_ constraints.

Five patients (2 prostate cases, 2 L_SAC_ cases and 1 H_SAC_ case) were randomly selected for optimized treatment plans with the new translated D_LEM|v_ constraints. The lower boundary values of the 95% CI were considered for D_LEM|v_ to account for uncertainties involved in the D_RBE_ translation process. Plan optimization was performed with Syngo and dose distributions were then exported to FRoG for D_MKM_ recalculation. Rectum D_MKM_VHs were then analyzed to verify their compliance with the original D_MKM|v_ constraints.

From D_MKM_ distributions, the FRoG generated EUD values to predict the late rectal toxicity NTCP, based upon the formulation reported by Fukahori et al. [16,29,30]. In particular, the median values for LKB parameters n, m and TD50 (0.035 (95% CI: 0.024–0.047), 0.10 (0.084–0.13) and 63.6 Gy(RBE) (61.8–65.4)) obtained for ≥G1 late rectal toxicity were selected to calculate the NTCP for the CNAO population.

**Figure 6:**
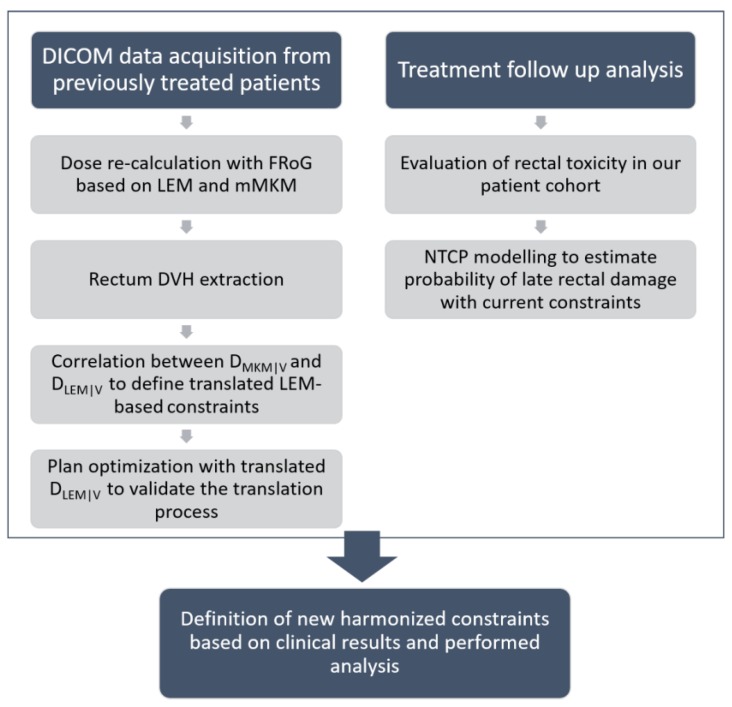
A scheme summarizing the study methodology.

A scheme summarizing the study methodology is presented in Figure 6, highlighting the two lines of investigation followed in this work. On one side, the conversion process of rectum dose distribution for 63 patients allowed the translation of D_RBE_ values between different RBE models. This part of the analysis provided a quantification of the potential for relaxation of LEM-based constraints. Concurrently, the follow-up of our pelvic patient cohort was analyzed to evaluate actual reported rectal toxicity. The recently published NTCP model from the Japanese NIRS group [16] was then applied to our dataset to estimate the probability of late rectal damage based on treatment outcomes registered with a longer follow-up. This second part of the study aimed at ensuring the absence of a significant toxicity rate with the currently applied constraints. New rectum dose constraints, to be confirmed prospectively using the clinical data, were finally defined for all the pelvic treatments scheduled in 16 fractions.

## 5. Conclusions

For the definition of new OAR dose constraints, a detailed RBE model translation analysis has been combined with the evaluation of treatment toxicity, based on long-term clinical follow-up data and normal tissue complication predictors, as estimated by other reference CIRT centers. With the clinical implementation of updated dose constraints, we expect an improvement of treatment quality by allowing a potentially higher dose delivery to the tumor, with no increase in healthy tissue toxicity. The methodology applied in this work was successfully followed for the optic pathways [15] and brainstem. Updating dose constraints for organs at risk in the treatment of pancreatic AdC tumors and other abdominal lesions (i.e., duodenum, stomach, small bowel and colon) is currently ongoing.

## Figures and Tables

**Table 1:** Rectum D_LEM|v_ and D_MKM|v_ (v: 20%, 10%, 5%, 1% of the rectum volume) for five patients’ treatment plans (two prostate AdC cases, two Sacral chordoma cases from the L_SAC_ group, and one case from the H_SAC_ group), optimized with translated D_LEM|v_ constraints. The old D_MKM|v_ and translated D_LEM|v_ constraints are reported in brackets in the corresponding column heading. The rectum volume of each patient is also presented. D_20%_ and D_10%_ were not applied for sacral chordoma plan optimization.

Case	Rectum Volume (cc)	D_LEM|20%_ (38.7 Gy(RBE))	D_MKM|20%_ (28.8 Gy(RBE))	D_LEM|10%_ (54.7 Gy(RBE))	D_MKM|10%_ (46.4 Gy(RBE))	D_LEM|5%_ (65.1 Gy(RBE))	D_MKM|5%_ (60.0 Gy(RBE))	D_LEM|1%_ (69.3 Gy(RBE))	D_MKM|1%_ (66.0 Gy(RBE))
Prostate 1	68.6	26.3	14.0	50.9	38.1	61.4	50.7	67.1	63.3
Prostate 2	58.3	37.2	23.6	54.8	41.8	61.1	49.3	64.5	55.0
L_SAC_ 1	146.2	47.2	35.1	59.9	50.8	63.8	56.3	67.9	62.7
L_SAC_ 2	53.5	25.6	11.7	47.7	32.7	59.8	49.7	67.2	60.5
H_SAC_ 1	86.0	48.6	37.0	58.8	49.4	63.3	54.8	67.7	60.5
